# Case report: Double-lung transplantation for Hermansky–Pudlak syndrome-associated pulmonary fibrosis and early-stage lung cancer

**DOI:** 10.3389/fimmu.2026.1684523

**Published:** 2026-03-24

**Authors:** Xiaotong Li, Liangxia Ding, Bin Li, Zhaolei Ding, Kun Wang, Junjuan Li, Xiaopeng Gao

**Affiliations:** 1 Department of Respiratory and Critical Care Medicine, WeiFang People’s Hospital, Shandong Second Medical University, Weifang, Shandong, China; 2 Department of Intravenous Medicine Dispensing, WeiFang People’s Hospital, Shandong Second Medical University, Weifang, Shandong, China; 3 Department of Orthopaedic Surgery, WeiFang People’s Hospital, Shandong Second Medical University, Weifang, Shandong, China

**Keywords:** Hermansky-Pudlak syndrome, lung cancer, lung transplantation, MUC5B mutation, pulmonary fibrosis

## Abstract

Lung transplantation for lung cancer remains exploratory but may benefit patients with concurrent end-stage lung disease. We present a 47-year-old Asian male with Hermansky-Pudlak syndrome-associated pulmonary fibrosis (HPS-PF), a congenital right-sided aortic arch, and a MUC5B mutation—an exceptionally rare case of multifactorial pulmonary fibrosis complicated by lung cancer. Preoperatively, he exhibited rapid functional decline. After bilateral lung transplantation, respiratory function and quality of life improved significantly, enabling a return to normal work within one month. At one-year follow-up, no tumor recurrence was observed. This case highlights potential indications for transplantation in lung cancer, the need for precise preoperative assessment, tailored postoperative therapy, and recurrence risk evaluation. It also emphasizes genetic factors in pulmonary fibrosis, suggesting gene therapy and editing as future therapeutic avenues.

## Introduction

Hermansky-Pudlak Syndrome (HPS), first described and named by Hermansky and Pudlak in 1959, is a rare autosomal recessive disorder characterized by cutaneous albinism, platelet dysfunction leading to hemorrhage, interstitial pulmonary fibrosis, and granulomatous colitis (1). The global incidence of HPS is estimated to be between 1 and 9 per million. The syndrome is more prevalent among Hispanics, with a reported incidence of approximately 1 in 18,000 in northwestern Puerto Rico (2). HPS is genetically heterogeneous and categorized into 10 subtypes based on mutations affecting the biogenesis of lysosome-associated organelle complexes or the adaptor protein (AP)-3 complex. These include BLOC-1 (HPS-7, HPS-8, and HPS-9), BLOC-2 (HPS-3, HPS-5, and HPS-6), BLOC-3 (HPS-1 and HPS-4), and AP-3 complex (HPS-2 and HPS-10). Pulmonary fibrosis is mainly associated with mutations in three HPS genes: HPS1, HPS2, and HPS4 (3). Most patients harboring HPS-1 mutations develop pulmonary fibrosis, which is often fatal (4). For end-stage HPS-related pulmonary fibrosis (HPS-PF), lung transplantation remains the definitive treatment (5).

Patients with interstitial lung disease (ILD), especially those with pulmonary fibrosis, have a higher risk of developing lung cancer (6). The survival of lung cancer patients with concurrent ILD is significantly shorter than that of patients with lung cancer alone (7). Lung transplantation is rarely performed in patients with lung cancer. Lung cancer accounts for only 0.1% of lung transplant indications over the past 20 years (8). Lung transplantation is often not recommended for patients with lung cancer; however, incidental detection of lung cancer may not significantly affect transplantation prognosis (9). A systematic review suggests that transplant patients with early-stage, incidentally resected non-small cell lung cancer (NSCLC) have survival rates comparable to those of certain cancer-free transplant patients (10). Therefore, selecting lung cancer patients who may benefit from lung transplantation remains a clinical challenge.

We report a rare case of double lung transplantation performed to treat HPS-PF in a patient with a congenital right-sided aortic arch and a mutation in the MUC5B gene. The primary focus of this report is the management of end-stage HPS-associated pulmonary fibrosis requiring urgent transplantation, with lung adenocarcinoma incidentally identified on explant pathology, raising important considerations regarding transplantation in patients with occult malignancy.

## Case description

A 47-year-old Asian male, married and working as a medical professional, was admitted to the hospital in March 2024 due to a two-year history of intermittent cough and chest tightness, which had worsened over the previous five months. Since January 2022, he had experienced a dry cough without any obvious trigger, accompanied by chest tightness after exercise. He denied rash, arthralgia, dry mouth or dry eyes, and did not report Raynaud’s phenomenon during this period. Five months prior to admission, his symptoms progressively worsened without a clear cause. His exercise tolerance decreased rapidly, and he began to experience chest tightness even at rest. He was first admitted to China-Japan Friendship Hospital, where genetic testing confirmed a diagnosis of Hermansky-Pudlak syndrome. Treatment with oral pirfenidone was initiated but proved ineffective, so he sought further management at our hospital.

**Figure 1:**
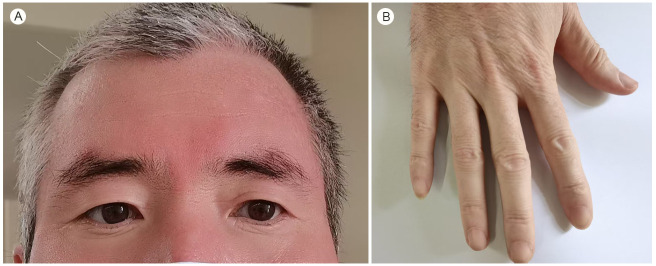
Facial manifestations of the patient: **(A)** Partial greying of the hair, black eyebrows, eyelashes and sweaty hairs on the hands. **(B)** Slight hypopigmentation of the face and hands.

Past medical history: albinism and second-degree visual impairment. Personal history: no history of smoking or alcohol consumption. Family history: parents deceased, no history of albinism. Reproductive history: father of two daughters, neither with albinism; spouse is healthy. Physical examination: the patient’s hair was partially white, while the eyebrows, eyelashes, and fine hair on the hands were black. Mild hypopigmentation of the face and hands was noted (**Figure 1**). On auscultation, coarse breath sounds were heard bilaterally, with Velcro-like crackles audible during inspiration in both lungs.

Laboratory tests: Routine blood, urine, and fecal tests, as well as cardiac enzymes, pro-BNP, liver function tests, anti-CCP antibody, rheumatoid factor, quantitative antinuclear antibody, antinuclear antibody profile, anti-vasculitis antibody, and anti-cardiolipin antibody were all within normal limits.

Coagulation tests showed a prolonged activated partial thromboplastin time (APTT) of 39.4 s (normal range: 21.2–34.6 s) and an elevated D-dimer level of 6.19 µg/mL (normal range 0.00 –0.55 µg/mL). Platelet aggregation function test: COL-180s aggregation rate 16.0% (normal range: 39–80%), COL-300s aggregation rate 7.9% (normal range: 50–84%), COL-maximal aggregation rate 17.2% (normal range: 52–84%), COL-300s mean aggregation rate 9.4% (normal range: 40.5–75.6%). Tumour markers: CEA 28.2 ng/mL, NSE 17.1 ng/mL, CA125 62.3 ng/mL. Blood gas analysis (FiO_2_ 37%): pH 7.45, PaO_2–_ 48 mmHg, PaCO_2–_ 40 mmHg, minimum oxygen saturation 85%. Pulmonary function testing and 6-minute walk test could not be completed due to poor tolerance.

**Figure 2:**
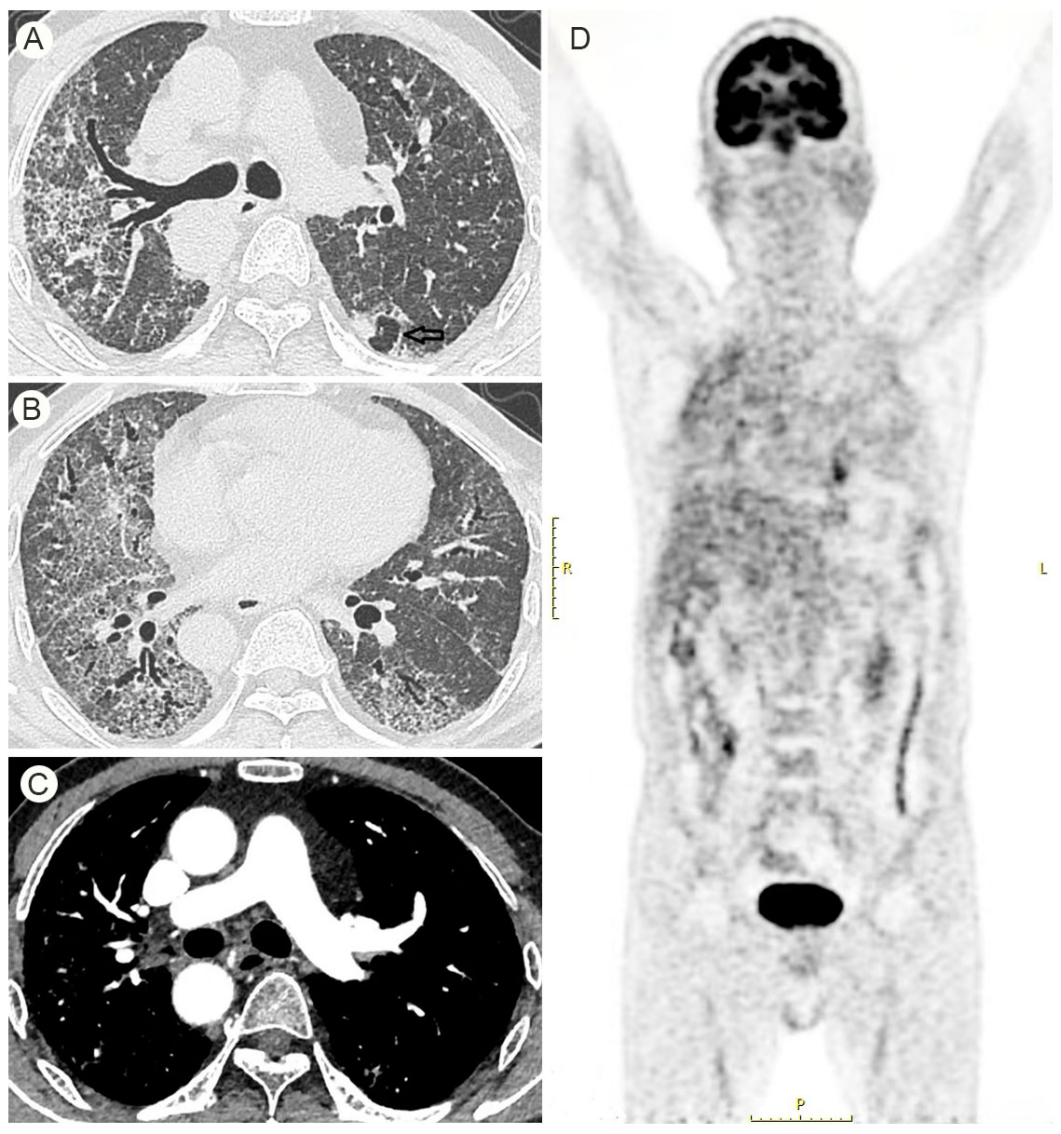
Preoperative HRCT signs: **(A, B)** diffuse ground-glass shadows in both lungs, dilated fine bronchioles, reticular shadows, striated lines and stretched bronchioles were seen in the proximal pleura, volume reduction in the lower lungs bilaterally. **(C)** Right aortic arch. **(D)** Foci of fibrosis in both lungs, foci of calcification in both lungs, localised thickening of the pleura in both lungs, inflammatory enlarged lymph nodes in the mediastinum.

High-resolution CT (HRCT) of the chest (**Figures 2A–C**) revealed a right-sided aortic arch, diffuse ground-glass opacities in both lungs, dilated small bronchioles, reticular shadows, striated lines, and stretched bronchioles adjacent to the pleura. There was also bilateral lower lung volume loss and thickening of the interlobar fissures. PET/CT (**Figure 2D**) demonstrated fibrotic foci and calcified lesions in both lungs, localised pleural thickening bilaterally, inflammatory enlargement of mediastinal lymph nodes, and a right-sided aortic arch. Genetic testing (**Supplementary Figure 1**) identified two heterozygous mutations in the *HPS1* gene: c.612delC, a frameshift deletion mutation, and c.936_938delinsTTTGTTTTTCAAGTTTGT, a deletion-insertion mutation. Additionally, two heterozygous mutations were found in the *MUC5B* gene: c.G7762A and c.C13223T, both of which are classified as missense mutations.

**Figure 3:**
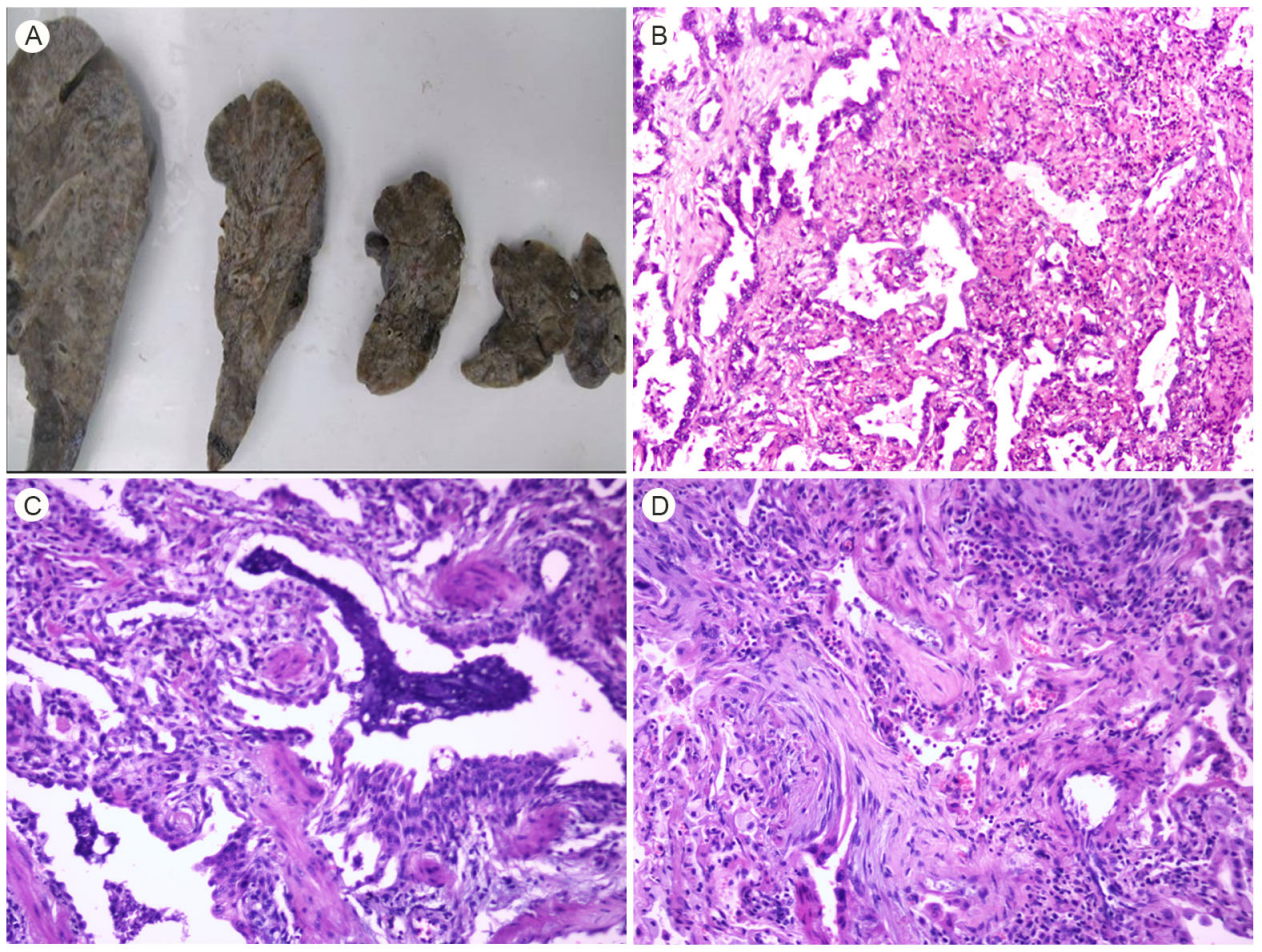
Gross and microscopic findings of the resected lungs. **(A)** Gross specimen: the pleural surface is rough and nodular with localized honeycombing. **(B)** Invasive poorly differentiated adenocarcinoma invading the pleura, with vascular tumor emboli but no perineural invasion (H&E staining, ×200). **(C)** Partially cystically dilated alveolar spaces and small bronchioles showing honeycomb-like changes (H&E staining, ×200). **(D)** Phagocytes and foamy macrophages accumulate in the alveolar lumen with foci of organization, consistent with organizing pneumonia changes (H&E staining, ×200).

**Figure 4:**
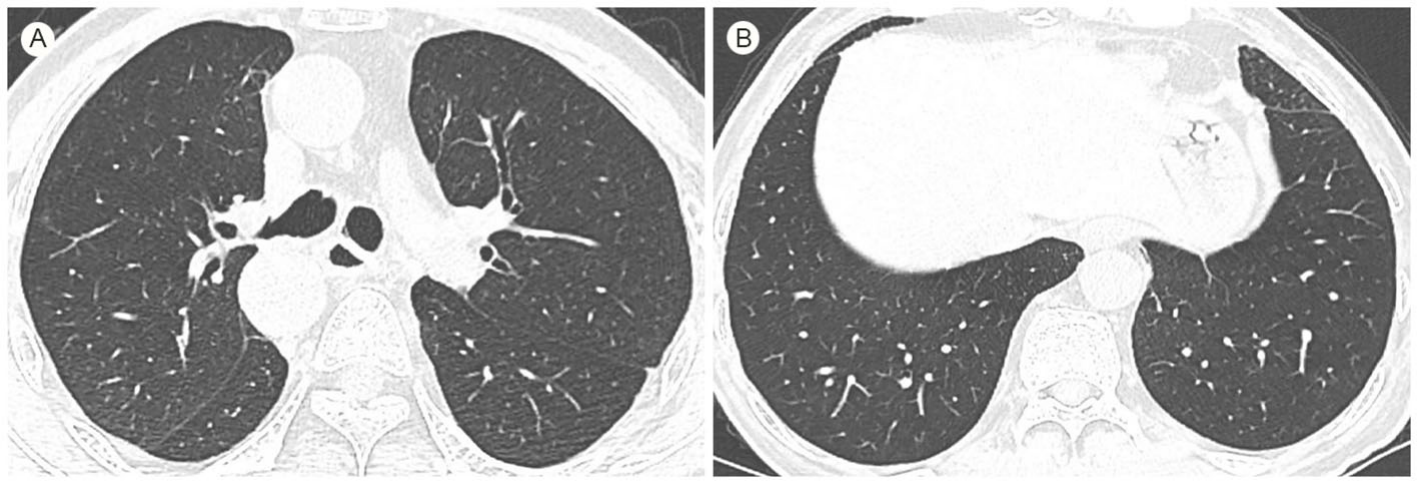
Postoperative HRCT at 3 month: **(A, B)** good translucency in both lungs.

The multi-disciplinary team (MDT) at the Lung Transplant Centre determined that the patient had end-stage interstitial lung disease (ILD) and recommended bilateral lung transplantation. On April 7, 2024, under general anaesthesia with preserved spontaneous respiration and veno-venous extracorporeal membrane oxygenation (VV-ECMO) support, the patient underwent bilateral lung transplantation and total pneumonectomy using lungs from an unrelated donor. Gross examination of the resected lung specimen (**Figure 3A**) showed a specimen measuring 18 × 15 × 3 cm. The interlobular fissure between the upper and lower lobes was clear. The visceral pleural surface was rough with multiple nodular protrusions. In the upper lobe, a firm area measuring 2.5 × 1.8 × 1.5 cm was located 1.2 cm from the pleural surface and 3.5 cm from the bronchial margin; this area showed local pleural retraction and crumpling. The remaining parenchyma of the upper lobe appeared fibrotic and collapsed. The lower lobe exhibited localised honeycombing with more severe lesions than in the upper lobe. The resected right lung measured 18 × 16 × 4 cm with well-defined interlobular fissures. Localized honeycombing was noted, with more severe changes in the upper lobe compared to the middle and lower lobes. Postoperative histopathology revealed invasive adenocarcinoma in the upper lobe of the left lung, poorly differentiated (approximately 75% of acinar and 25% complex glandular pattern), measuring 2.5 × 1.8 × 1.5 cm, invading the pleura, with vascular tumor emboli and no definite perineural invasion (**Figure 3B**). The remaining lung tissue showed patchy interstitial fibrosis with varying degrees of severity bilaterally (**Figures 3C, D**). Metastatic carcinoma was identified in peribronchial lymph nodes (2/19 in the left lung and 0/19 in the right lung). According to the 8th edition of the American Joint Committee on Cancer (AJCC) staging system, the tumor was classified as pT2aN1M0, corresponding to stage IIB disease. Postoperative tumor tissue genetic analysis (**Supplementary Figure 2**) detected mutations in *NRAS* and *TP53*. Tacrolimus combined with methylprednisolone was administered postoperatively to prevent rejection, which did not occur. The patient was transferred to the general ward on postoperative day 4 and was discharged on postoperative day 14. Follow-up fibreoptic bronchoscopy one month after surgery showed good anastomotic healing. One month postoperatively, the patient had returned to normal life and work with an activity level of up to 10,000 steps per day. Circulating tumor cell (CTC) testing revealed three CTCs. One-year HRCT (**Figure 4**) showed good postoperative bilateral lung anastomosis, clear bronchovascular bundles, and no abnormal density foci in the lungs. Lung function reported forced expiratory volume in one second (FEV1) of 3.05L.

## Discussion

Lung transplantation remains a controversial option for patients with lung cancer. From 1995 to 2018, only 0.1% of lung transplants were performed in patients with this diagnosis (8). This patient had no family history of lung cancer, and preoperative CT and PET/CT scans did not reveal any obvious primary lung malignancy. The preoperative carcinoembryonic antigen (CEA) level was 28.2 ng/mL, slightly above the reference range, which may have been attributable to the underlying pulmonary fibrosis. A retrospective review identified a suspicious lesion in the upper lobe of the left lung prior to transplantation (indicated by the arrow in **Figure 2A**). However, this lesion was not sufficiently recognized before surgery. Small lung cancer nodules are often difficult to detect in the presence of diffuse fibrotic changes and may easily be mistaken for fibrotic foci (11). Even with thorough preoperative imaging, the incidence of incidental malignancies in explanted lung tissue is estimated at around 2.2%, which is considerably higher than the rate of lung cancer as an indication for lung transplantation (12).

This observation raises an important clinical question: would lung transplantation have been pursued if the malignancy had been clearly identified preoperatively? In the present case, the indication for transplantation was rapidly progressive end-stage HPS-associated pulmonary fibrosis with severe hypoxemia and poor functional tolerance. The lung tumor was not definitively recognized before surgery. If detected during pre-transplant evaluation, the decision would have required a comprehensive multidisciplinary discussion weighing the fatal trajectory of HPS-PF against the oncologic risk under immunosuppression.

The main concern with lung transplantation for patients with lung cancer is that postoperative immunosuppressive therapy increases the risk of cancer recurrence. In addition, these patients often undergo extensive preoperative therapy, which may elevate the risk of postoperative complications (13). However, a single-center retrospective study demonstrated that lung transplant recipients with incidentally detected stage I NSCLC had 1- and 5-year survival rates comparable to those of recipients without cancer (14). A joint analysis of the Scientific Registry of Transplant Recipients and the National Cancer Database showed that clinical outcomes for lung transplant patients with stage I/II NSCLC or any stage of neuroendocrine carcinoma were similar to those of patients without malignancy, whereas outcomes were poorer in patients with stage III/IV NSCLC (15). Case series and analyses of Organ Procurement and Transplantation Network (OPTN) registry data suggest that double-lung transplantation (DLT) may improve survival in patients with refractory bilateral lung cancer. (16) Similarly, the one-year survival rate for patients with incidentally detected lung cancer at the time of transplantation remains encouraging (17).

The appropriateness of lung transplantation in patients with lung malignancy remains controversial. While advanced-stage disease is generally considered a contraindication due to the high recurrence risk under lifelong immunosuppression, emerging evidence suggests that carefully selected patients with localized or completely resectable tumors may achieve acceptable outcomes (14). In this case, postoperative staging revealed pT2aN1M0 (stage IIB) disease with complete surgical resection through pneumonectomy. Although nodal involvement was present (N1), no distant metastasis was detected, and no recurrence has been observed at one-year follow-up. Nevertheless, long-term oncologic outcomes remain uncertain and require continued surveillance.

The use of immunosuppressive therapy after transplantation increases the risk of cancer recurrence. Early detection of tumours following transplantation also remains a significant challenge. For early detection, chest imaging is recommended at least every three months in patients at high risk for lung cancer. When indicated, combined PET and chest CT may assist in evaluating suspected malignancies (18). Dynamic monitoring of tumor markers may help detect recurrence early, although limited sensitivity and specificity constrain their utility. Circulating extracellular nucleic acids (cell-free DNA; cfDNA) and circulating tumor DNA (ctDNA) can be isolated from peripheral blood; cfDNA includes DNA fragments that may or may not originate from tumors, whereas ctDNA is specifically tumor-derived. Circulating tumor cells (CTCs) are intact cells shed into the bloodstream by the tumor. Both cfDNA/ctDNA and CTCs are increasingly being explored as prognostic and monitoring tools after surgery (19).

The optimal management of patients who develop lung cancer after transplantation remains unclear. Genomic profiling is routinely performed for non-small cell lung cancer (NSCLC), and a high tumor mutational burden (TMB) may support consideration of immunotherapy (20), although this approach poses additional risks in lung transplant recipients. Chemotherapy and radiotherapy are generally unsuitable for these patients because of their immunosuppressed state, and in some cases, these treatments have been associated with severe acute complications (21). Targeted therapies may provide alternative options, but robust clinical data are still lacking. Overall, the treatment of lung cancer following transplantation remains a significant clinical challenge.

HPS is a rare autosomal recessive disorder involving defective biogenesis of lysosome-related organelles. Among its ten genetic subtypes, HPS-1, HPS-2, and HPS-4 are most commonly associated with pulmonary fibrosis. Pulmonary fibrosis related to HPS-2 typically occurs in children, whereas fibrosis due to HPS-1 or HPS-4 more often develops in middle-aged adults (1). The present case involved HPS-1–related pulmonary fibrosis with adult onset and rapid progression. Genetic analysis also revealed a coexisting MUC5B mutation, which is known to promote the development of pulmonary fibrosis (22). However, it remains unclear whether the coexistence of HPS-1 and MUC5B mutations has a synergistic effect on disease onset or severity.

Light microscopy of lung tissue from HPS-related pulmonary fibrosis reveals foamy alveolar macrophages and hyperplastic type II alveolar epithelial cells. Electron microscopy demonstrates enlarged type II alveolar epithelial cells containing abundant lamellar bodies. In murine models of HPS-PF, a high accumulation of surfactant-like material has also been observed in alveolar epithelial cells (23). However, studies have indicated that long-term treatment with pirfenidone may offer clinical benefits for HPS-PF (24), and antifibrotic drugs may have a potential role as second-line therapy for patients with progressive non-IPF interstitial lung diseases (25). Currently, the U.S. Food and Drug Administration has not approved pirfenidone for the treatment of HPS-related pulmonary fibrosis. Lung transplantation remains the definitive treatment for end-stage HPS-PF, and recurrence of pulmonary fibrosis after transplantation has not been reported (26). Lentiviral-mediated gene transfer has been shown to restore HPS1 gene expression and function in patient-derived dermal melanocytes (27), while transgenic epithelial-specific correction of HPS defects can significantly attenuate bleomycin-induced alveolar epithelial cell apoptosis, fibrosis susceptibility, and macrophage activation (23). These findings suggest that gene therapy and gene editing may be promising future strategies for treating HPS pulmonary fibrosis. Anandamide (arachidonoyl ethanolamide, AEA) has also been proposed as a potential diagnostic and prognostic biomarker for pulmonary fibrosis, including HPS-PF (28). Such novel biomarkers may help optimize patient prognosis based on individual genetic profiles and facilitate earlier, more accurate detection of HPS-PF.

## Conclusion

The use of lung transplantation in patients with lung cancer remains controversial. Limited evidence and experience regarding lung transplantation for thoracic malignancies has led many transplant centers to be reluctant to perform this procedure. This hesitation has restricted the broader clinical application of lung transplantation in lung cancer patients. The case demonstrates that lung transplantation may be feasible in highly selected patients with end-stage lung disease and incidentally detected or localized malignancy. However, it does not support routine expansion of transplantation indications to lung cancer patients. Transplant centers should carefully select appropriate candidates through multidisciplinary discussions, considering the clinical stage, pathological type, and comorbidities, to provide individualized and precise treatment.

## Data Availability

The original contributions presented in the study are included in the article/**Supplementary Material**. Further inquiries can be directed to the corresponding author.

## Supplementary material

Supplementary Figure 1 Genetic testing report: 2 mutations in HPS1 gene, 2 mutations in MUC5B gene.

Supplementary Figure 2 Genetic testing: NRAS, TP53 mutations.

## References

[1] Yokoyama T Gochuico BR . Hermansky-Pudlak syndrome pulmonary fibrosis: a rare inherited interstitial lung disease. Eur Respir Rev. (2021) 30:200193. doi: 10.1183/16000617.0193-2020. PMID: 33536261 PMC9488956

[2] Huizing M Malicdan MCV Wang JA Pri-Chen H Hess RA Fischer R . Hermansky-Pudlak syndrome: Mutation update. Hum Mutat. (2020) 41:543–80. doi: 10.1002/humu.23968. PMID: 31898847 PMC8175076

[3] Hu X Wei Z Wu Y Zhao M Zhou L Lin Q . Pathogenesis and therapy of Hermansky-Pudlak syndrome (HPS)-associated pulmonary fibrosis. Int J Mol Sci. (2024) 25:11270. doi: 10.3390/ijms252011270. PMID: 39457053 PMC11508683

[4] Brantly M Avila NA Shotelersuk V Lucero C Huizing M Gahl WA . Pulmonary function and high-resolution CT findings in patients with an inherited form of pulmonary fibrosis, Hermansky-Pudlak syndrome, due to mutations in HPS-1. Chest. (2000) 117:129–36. doi: 10.1378/chest.117.1.129. PMID: 10631210

[5] Benvenuto L Qayum S Kim H Robbins H Shah L Dimango A . Lung transplantation for pulmonary fibrosis associated with Hermansky-Pudlak syndrome. A single-center experience. Transplant Direct. (2022) 8:e1303. doi: 10.1097/txd.0000000000001303. PMID: 35350109 PMC8947604

[6] Frank AJ Dagogo-Jack I Dobre IA Tait S Schumacher L Fintelmann FJ . Management of lung cancer in the patient with interstitial lung disease. Oncologist. (2023) 28:12–22. doi: 10.1093/oncolo/oyac226. PMID: 36426803 PMC9847545

[7] Alomaish H Ung Y Wang S Tyrrell PN Zahra SA Oikonomou A . Survival analysis in lung cancer patients with interstitial lung disease. PloS One. (2021) 16:e255375. doi: 10.1371/journal.pone.0255375. PMID: 34492020 PMC8423282

[8] Chambers DC Cherikh WS Harhay MO Hayes D Jr Hsich E Khush KK . The International Thoracic Organ Transplant Registry of the International Society for Heart and Lung Transplantation: Thirty-sixth adult lung and heart-lung transplantation report-2019; focus theme: Donor and recipient size match. J Heart Lung Transplant. (2019) 38:1042–55. doi: 10.1016/j.healun.2019.08.001. PMID: 31548030 PMC6816340

[9] Machuca TN Keshavjee S . Transplantation for lung cancer. Curr Opin Organ Transplant. (2012) 17:479–84. doi: 10.1097/mot.0b013e328357fff6. PMID: 22907541

[10] Elsolh B Bayat Z Lyu D Lin J Wakeam E . Lung transplantation for lung cancer: a systematic review of the literature. J Heart Lung Transplant. (2023) 42:1425–36. doi: 10.1016/j.healun.2023.05.011. PMID: 37253398

[11] Hendriks LE Drent M van Haren EH Verschakelen JA Verleden GM . Lung cancer in idiopathic pulmonary fibrosis patients diagnosed during or after lung transplantation. Respir Med Case Rep. (2012) 5:37–9. doi: 10.1016/j.rmedc.2011.10.003. PMID: 26029585 PMC3920419

[12] Amratia DA Hunt WR Neujahr D Veeraraghavan S . Incidentally detected Malignancies in lung transplant explants. Transplant Direct. (2019) 5:e503. doi: 10.1097/txd.0000000000000947. PMID: 31773056 PMC6831122

[13] Shtraichman O Ahya VN . Malignancy after lung transplantation. Ann Transl Med. (2020) 8:416. doi: 10.21037/atm.2020.02.126. PMID: 32355860 PMC7186714

[14] Spies CS Ochoa TN Pontula A Harris CS Snyder LD Pavlisko EN . With comparable outcomes, should early-stage lung cancer be a contraindication to lung transplant? Ann Thorac Surg. (2024) 118:261–7. doi: 10.1016/j.athoracsur.2023.09.002. PMID: 37704001

[15] Rebernick RJ Martinez JD De Perrot M Cypel M Keshavjee S Reddy RM . Prognostic implications of lung cancers incidentally identified on explant: a joint study of the Scientific Registry of Transplant Recipients and the National Cancer Database. Am J Transplant. (2025) 25:814–24. doi: 10.1016/j.ajt.2024.11.014. PMID: 39557123

[16] Lee J Aguilera CM Yu J Chung LI Bharat A Chae YK . Survival outcomes after double-lung transplantation for refractory lung-limited cancers and incidence of post-transplant lung cancer. Ann Transplant. (2023) 28:e941301. doi: 10.12659/aot.941301. PMID: 38050347 PMC10709990

[17] Bouchez C Medraoui C Cazes A Khalil A Jebrak G Mal H . Prognosis of incidental lung cancer in lung transplant candidates. Respir Med Res. (2024) 87:101146. doi: 10.1016/j.resmer.2024.101146. PMID: 39689665

[18] Choi YJ Kim SY Park MS Lee JG Paik HC Lee SH . Incidental lung cancer of explanted lungs from lung transplant recipients: incidence, characteristics, and 5-year survival. Yonsei Med J. (2020) 61:958–64. doi: 10.3349/ymj.2020.61.11.958. PMID: 33107239 PMC7593106

[19] Nikanjam M Kato S Kurzrock R . Liquid biopsy: current technology and clinical applications. J Hematol Oncol. (2022) 15:131. doi: 10.1186/s13045-022-01351-y. PMID: 36096847 PMC9465933

[20] Shi Y Lei Y Liu L Zhang S Wang W Zhao J . Integration of comprehensive genomic profiling, tumor mutational burden, and PD-L1 expression to identify novel biomarkers of immunotherapy in non-small cell lung cancer. Cancer Med. (2021) 10:2216–31. doi: 10.1002/cam4.3649. PMID: 33655698 PMC7982619

[21] Tseng SC Gagne SM Hatabu H Lin G Sholl LM Nishino M . Lung cancer in lung transplant recipients: clinical, radiologic, and pathologic characteristics and treatment outcome. J Comput Assist Tomogr. (2023) 47:590–7. doi: 10.1097/rct.0000000000001466. PMID: 36944140 PMC10363202

[22] Mathai SK Humphries S Kropski JA Blackwell TS Powers J Walts AD . MUC5B variant is associated with visually and quantitatively detected preclinical pulmonary fibrosis. Thorax. (2019) 74:1131–9. doi: 10.1136/thoraxjnl-2018-212430. PMID: 31558622 PMC7535073

[23] Young LR Gulleman PM Bridges JP Weaver TE Deutsch GH Blackwell TS . The alveolar epithelium determines susceptibility to lung fibrosis in Hermansky-Pudlak syndrome. Am J Respir Crit Care Med. (2012) 186:1014–25. doi: 10.1164/rccm.201207-1206oc. PMID: 23043085 PMC3530211

[24] O'Brien KJ Introne WJ Akal O Akal T Barbu A McGowan MP . Prolonged treatment with open-label pirfenidone in Hermansky-Pudlak syndrome pulmonary fibrosis. Mol Genet Metab. (2018) 125:168–73. doi: 10.1016/j.ymgme.2018.07.012, PMID: 30055995

[25] George PM Spagnolo P Kreuter M Altinisik G Bonifazi M Martinez FJ . Progressive fibrosing interstitial lung disease: clinical uncertainties, consensus recommendations, and research priorities. Lancet Respir Med. (2020) 8:925–34. doi: 10.1016/s2213-2600(20)30355-6. PMID: 32890499

[26] El-Chemaly S O'Brien KJ Nathan SD Weinhouse GL Goldberg HJ Connors JM . Clinical management and outcomes of patients with Hermansky-Pudlak syndrome pulmonary fibrosis evaluated for lung transplantation. PloS One. (2018) 13:e194193. doi: 10.1371/journal.pone.0194193. PMID: 29547626 PMC5856338

[27] Ikawa Y Hess R Dorward H Cullinane AR Huizing M Gochuico BR . *In vitro* functional correction of Hermansky-Pudlak syndrome type-1 by lentiviral-mediated gene transfer. Mol Genet Metab. (2015) 114:62–5. doi: 10.1016/j.ymgme.2014.11.006. PMID: 25468649 PMC4279856

[28] Cinar R Basu A Arif M Park JK Zawatsky CN Zuo BLG . Anandamide is an early blood biomarker of Hermansky-Pudlak syndrome pulmonary fibrosis. medRxiv. (2024). doi: 10.1101/2024.05.16.24307300, PMID: 39841973 PMC12005043

