# Supplementary figures and images for: Case report: Double-lung transplantation for Hermansky–Pudlak syndrome-associated pulmonary fibrosis and early-stage lung cancer
Source: Front Immunol. 2026 Mar 24;17:1684523. doi: 10.3389/fimmu.2026.1684523 (PMC13053257; doi:10.3389/fimmu.2026.1684523)

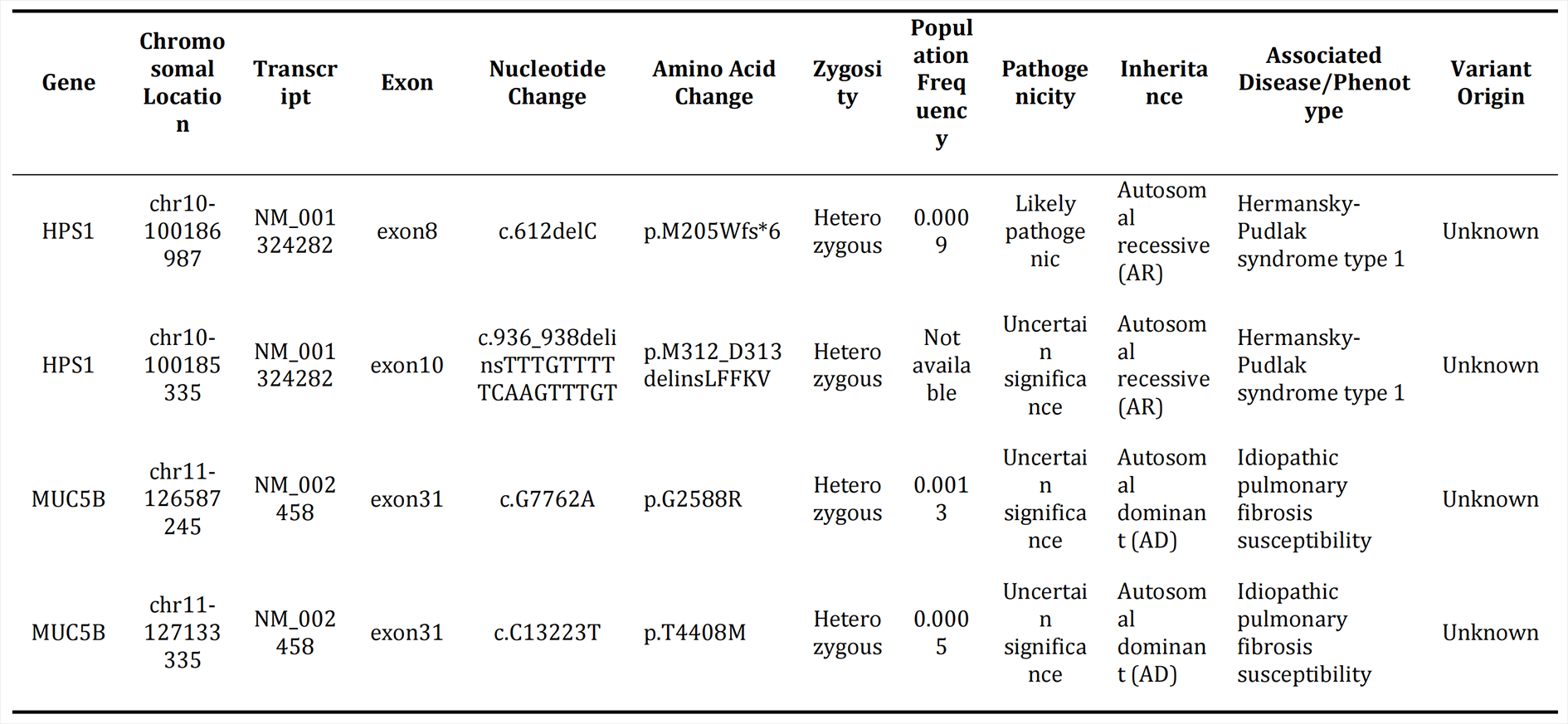

Supplement: Supplementary Figure 1 — Genetic testing report: 2 mutations in HPS1 gene, 2 mutations in MUC5B gene. [file Image1.tif]

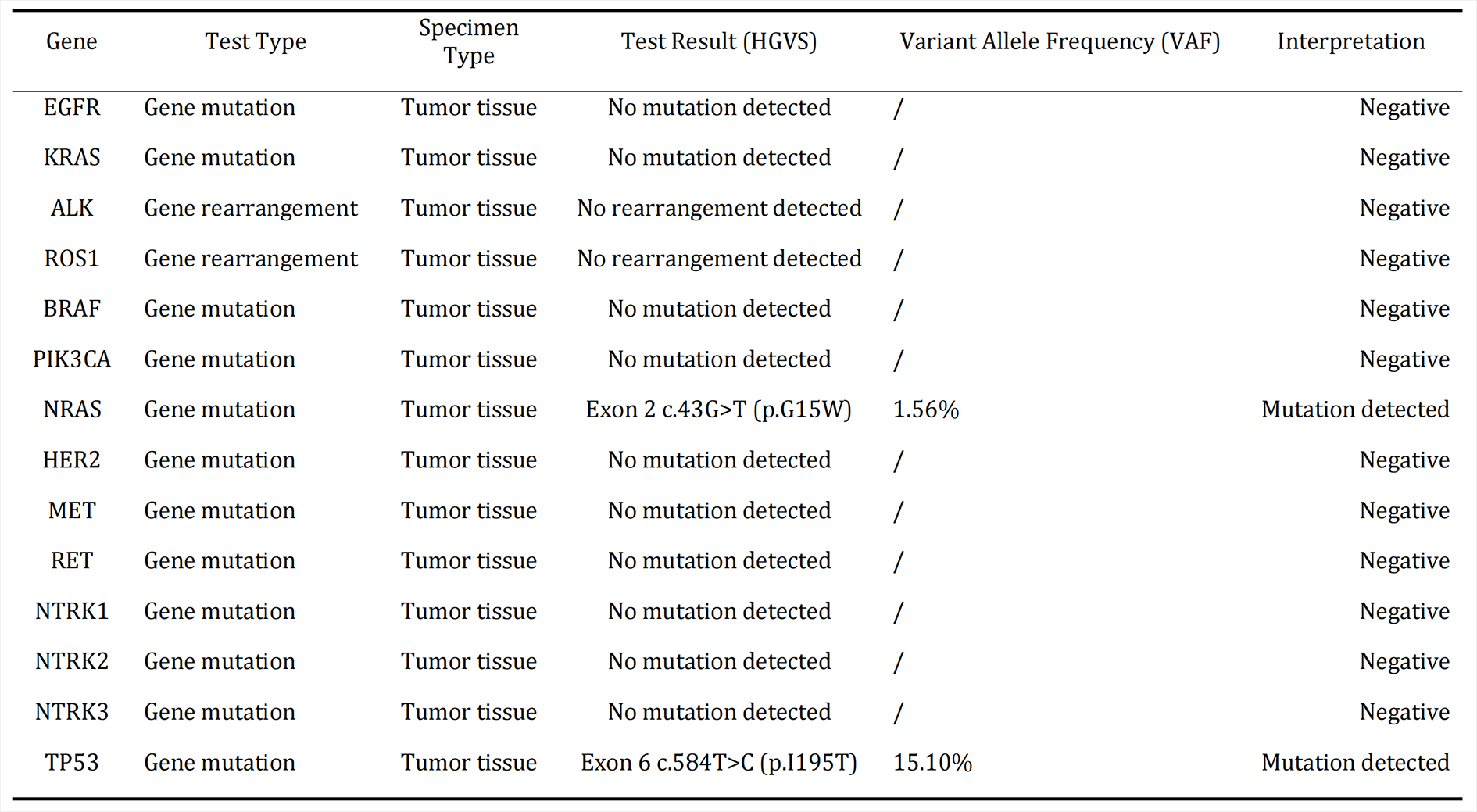

Supplement: Supplementary Figure 2 — Genetic testing: NRAS, TP53 mutations. [file Image2.tif]
